# Application of a Dopa Derivative for the Formation of Gels in the Presence of Commercial Surfactants

**DOI:** 10.3390/gels11050320

**Published:** 2025-04-25

**Authors:** Sofia Chinelli, Fabia Cenciarelli, Demetra Giuri, Claudia Tomasini

**Affiliations:** Dipartimento di Chimica Giacomo Ciamician, Università di Bologna, Via Piero Gobetti, 85, 40129 Bologna, Italy; sofia.chinelli2@unibo.it (S.C.); fabia.cenciarelli2@unibo.it (F.C.)

**Keywords:** anionic surfactant, gels, low molecular weight gelator, rheology, sustainable cosmetics

## Abstract

Cosmetic formulations are complex mixtures of ingredients that must fulfill several requirements. One of the challenges of the cosmetic industry is to find natural alternatives to replace synthetic polymers, preserving desirable sensory characteristics. The aim of this work is to induce the formation of gels, by replacing synthetic polymers with a low-molecular-weight gelator (LMWG), a small molecule able to self-assemble and form supramolecular networks. The impact of low-molecular-weight gelators on the environment is reduced as they are highly biodegradable. Thus, the behavior of solutions containing Boc-L-Dopa(Bn)_2_-OH, an LMWG, together with ten different anionic surfactants, was studied to understand if the LMWG may act as a rheological modifier by increasing the viscosity of the formulation or forming gels with these ingredients. An amphoteric surfactant, cocamidopropyl betaine (CAPB), often used to increase cleansing gentleness, was also added to the solutions to better mimic a cosmetic formulation. In most cases, the addition of the gelator at only a 1% *w*/*v* concentration induces the gelification or an increase in the viscosity of the solutions, thus showing that this molecule is also able to self-assemble in complex mixtures.

## 1. Introduction

Cosmetic formulations, encompassing solutions, emulsions, and solids, are designed for diverse body care applications, ranging from shampoos and conditioners to face and body creams. These products must meet several critical requirements. First and foremost, they must be non-toxic and effective for their intended use. Beyond safety and efficacy, the cosmetic industry prioritizes formulations that offer a pleasant skin feel for the consumer. Furthermore, there is a growing demand for “green” and eco-friendly products, emphasizing sustainable ingredients and environmentally conscious manufacturing processes. This includes considerations like biodegradability, reduced environmental impact, and the use of renewable resources [1,2]. Thus, the use of sustainable and eco-friendly ingredients is mandatory, but the preservation of the desired properties of the final product is challenging. One of the major concerns is synthetic polymers, which are usually employed as thickeners and rheological modifiers, increasing the viscosity of products and improving their texture. One of the challenges of the cosmetic industry is to find natural alternatives to replace these non-eco-friendly ingredients [3,4], preserving the desirable sensory characteristics.

To find sustainable replacements for synthetic polymers, several strategies are being explored. One approach is using natural polymers, such as polysaccharides [5,6], either individually or in mixtures. While using naturally derived polymers like polysaccharides is a logical step toward sustainability, this pathway signifies a significant hurdle. Matching the performance characteristics, specifically the thickening and gelling properties, of well-established synthetic polymers remains a challenge. This suggests that while polysaccharides offer a bio-based solution, they may not yet be able to fully replicate the desired functionality in many applications. Further research and modification of these natural polymers are likely needed.

Another promising avenue involves small, self-assembling molecules (called low-molecular-weight gelators, LMGWs) [7,8] that form fibrous networks capable of trapping large amounts of solvent, thus functioning as thickeners and gelling agents. This approach presents a more innovative and less explored alternative. LMWGs are small molecules that can self-assemble into three-dimensional networks within a solvent. This self-assembly is driven by non-covalent interactions, such as hydrogen bonds, van der Waals forces, and π–π stacking. These networks can trap substantial amounts of solvent, effectively thickening the material and creating gels. The mechanical properties of the obtained material can be assessed using rheology through a range of measurements that provide information on the formed network [9]. LMWGs are relatively underutilized in cosmetics, suggesting a significant potential for future development and applications [10,11]. 

LMWGs hold additional advantages as they offer a high degree of flexibility. Researchers can modify their chemical structure and manipulate the external conditions (the solvent, concentration, and trigger) to fine-tune the gel properties. This control allows for the design of LMWGs tailored to specific applications and desired sensory attributes. Moreover, the design of LMWGs often focuses on incorporating specific molecular groups (moieties and functional groups) that facilitate the non-covalent interactions necessary for self-assembly and gel formation.

Finally, a critical aspect of LMWG gelation is the control of solubility. The gelator is typically designed to be partially soluble in the chosen solvent. A trigger (e.g., a change in pH) is then used to reduce the solubility, causing the LMWG molecules to aggregate and form the gel network [12,13]. The passage provides a specific example of a pH-change method, where a base is used to initially dissolve a carboxylic acid-containing LMWG, and then an acid is added to reduce the pH below the pK_a_ of the gelator, thus indicating the protonation and the self-assembly of the gelator and consequently the solution gelation [14,15,16].

In essence, LMWGs represent a promising, albeit under-explored, avenue for developing sustainable alternatives to synthetic polymers in cosmetics and potentially other fields. Their tunability, self-assembly mechanism, and the ability to trigger gelation through changes in external conditions offer significant advantages for designing materials with tailored properties. The fact that they are relatively underutilized suggests a rich area for future research and development.

LMWGs can often be obtained by short amino acid sequences, even just dipeptides, or from single amino acids variously derivatized, for example, with fatty acids [17,18,19]. In the last case, their structure resembles exactly that of surfactants: a hydrophilic head (the amino acid moiety, often with the carboxylic acid group free and charged) and a long hydrophobic tail (the fatty acid) [20,21]. Some examples have already reported the ability of such structures to behave as gelators or at least viscosity enhancers, alone [22,23,24,25,26,27] and in combination with polymers [28,29,30]. Other works have also shown the beneficial self-assembly of surfactants with LMWGs as an approach to the formation of complex architectures, such as micelles or vesicles embedded in a gel matrix, or even interpenetrating networks [31,32,33,34]. These compounds offer the possibility to have LMWGs that are easily synthesized, biocompatible, biodegradable, non-toxic for the environment, and sometimes already tested and used in commercially available products [35,36,37]. In our work, we tested the ability of ten surfactants already on the market to form gels through self-assembly at a final pH of 5, as this is the physiological pH of the human skin and scalp. As most surfactants are not gelators, we added (*S*)-*N*-(tert-butyloxycarbonyl)-3,4-bis(benzyloxy)-phenylalanine (Boc-L-Dopa(Bn)_2_-OH), one of our previously reported LMWGs, to investigate the possibility of forming gels also with these ingredients. Indeed, Boc-L-Dopa(Bn)_2_-OH was chosen for these trials due to its ability to self-assemble in the presence of a variety of additives and species (crystals [38], other peptides [39], antioxidants [40], and fragrances [41]), proving to be a very robust and, at the same time, biocompatible [42] gelator.

Since anionic surfactants are quite aggressive [43,44], we studied the behavior of mixtures containing also cocamidopropyl betaine (CAPB), as this ingredient is added very often to commercial cosmetics to increase cleansing gentleness as it is mild on the skin [45].

## 2. Results and Discussion

**Figure 1:**
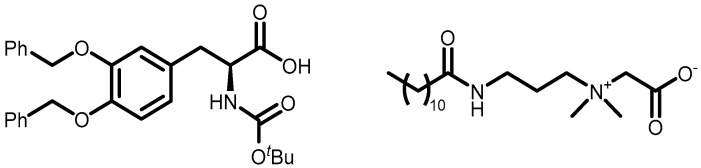
Chemical structure of Boc-L-Dopa(Bn)_2_-OH (**left**) and CAPB (**right**).

The aim of this work is to study the formation of gels from solutions containing commercial samples of the surfactants listed in Table 1, Boc-L-Dopa(Bn)_2_-OH, and CAPB (Figure 1). Ten different anionic surfactants were chosen as they represent the main classes of primary anionic surfactants often used in cosmetic formulations and allow for a relatively wide range of concentrations within their specification limits. The chemical structures of the main components of each surfactant are reported in Table 1, as most commercial surfactants contain small amounts of other components holding fatty acids with different chain lengths (**C12, C14, and C16**). Each surfactant (**1**–**10**) has a specific concentration range. For this reason, different amounts were withdrawn with a pipette or weighed in an analytical balance to achieve 10% *w*/*v* active matter in solution in any case. The average value of the active matter and the initial pH values are reported in Table S1.

Surfactants **1**–**4** are N-substituted Amino Acid Surfactants (AASs): Sodium Cocoyl Alaninate (**1**), Sodium Cocoyl Glycinate (**2**), Sodium Lauroyl Glutamate (**3**), and Sodium Lauroyl Sarcosinate (**4**). These molecules possess a carboxylate group, which makes them sensitive to the pH of the formulations, as the average pK_a_ of carboxylic acid is around **4**–**5**. In addition, surfactants **1**–**3** possess a secondary amide group that enables them to form hydrogen bonding.

The other six selected anionic surfactants contain a sulphonate or a sulphate group, which requires more extreme pH values to be protonated, making them less affected by pH modification as the pK_a_ of the corresponding acids is lower than −5. The sulphonates are Sodium Methyl Cocoyl Taurate (**5**), Sodium Methyl 2-Sulfolaurate (**6**), Sodium Lauroyl Methyl Isethionate (**7**), Disodium Laureth Sulfosuccinate (**9**), and Sodium **C14**–**16** Olefin Sulfonate (**10**). The sulphate is Magnesium Laureth Sulfate (**8**).

**Figure 2:**
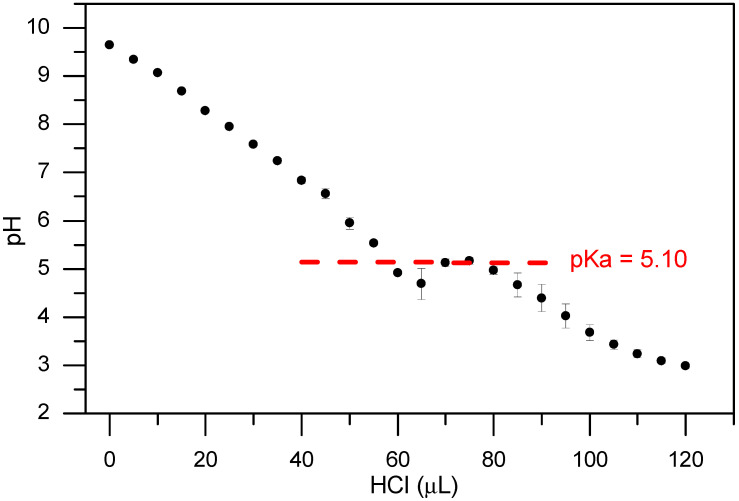
Titration curve (pH versus mol of added HCl 0.1 M) of Boc-L-Dopa(Bn)_2_-OH 0.2% *w*/*v*). The experiments were repeated in triplicate and the results are expressed as mean ± standard deviation. Black circles: titration curve; red line: highlight of the pK_a_ plateau.

The precise analysis of the pK_a_ of the surfactant is affected by the fact that they are commercial mixtures in solution, so their pK_a_ was not measured. In contrast, we could precisely measure the pK_a_ of Boc-L-Dopa(Bn)_2_-OH as it is a single component. The analysis was performed in a 0.2% concentration, where the molecule is fully soluble given a pK_a_ = 5.1. The result is reported in Figure 2.

Ten different samples (**1A**–**10A**) were prepared using the ten surfactants under condition A: the surfactant (10% *w*/*v*) and water were mixed with a magnetic stirrer for 15 min, then lactic acid (45% *w*/*v*) was added to reach a final pH of 5 while mixing with a magnetic stirrer. This pH was chosen because it is compatible with most cosmetic formulations and allows the protonation of carboxylic acids, thus favoring the molecules’ self-assembly and the consequent mixture gelation. L-Lactic acid was chosen because it is completely water soluble, cheap, biocompatible, and often used in cosmetic products [46,47].

Then, we prepared ten additional samples (**1B**–**10B**) under condition B: the surfactant (10% *w*/*v*), Boc-L-Dopa(Bn)_2_-OH (1% *w*/*v*), and water were mixed with a magnetic stirrer for 15 min, then lactic acid 45% *w*/*v* was added to adjust pH to 5.

Finally, another set of tests was performed under condition C, by using 10% *w*/*v* of anionic surfactant active matter together with Boc-L-Dopa(Bn)_2_-OH (1% *w*/*v*) and cocamidopropyl betaine (4.5% *w*/*v*) (samples **1C**-**10C**).

**Figure 3:**
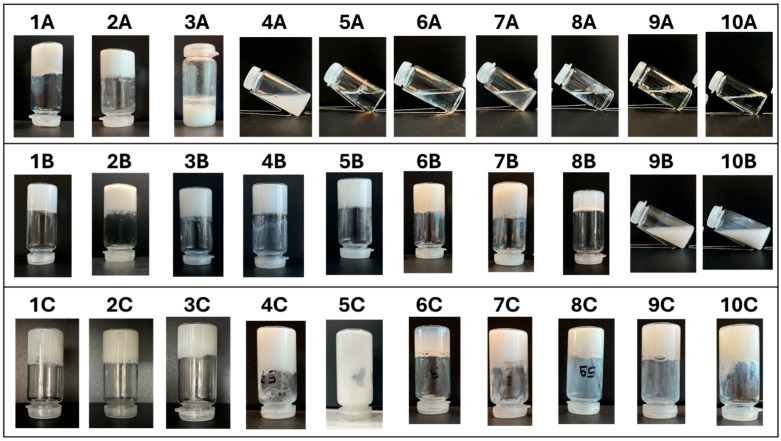
Photographs of the 30 samples obtained by gelation procedures **A**, **B**, and **C** with the ten surfactants studied in this work. The names of the samples are reported above the images.

Under condition **A**, samples **1A** and **2A** formed a gel at pH 5, while **3A** formed a precipitate (Figure 3). In any case, a modification of the mixture was obtained at pH 5 due to the protonation of the carboxylate. A totally different outcome was obtained with surfactants **4**–**10**. In all these cases, the surfactants had much lower pK_a_ (about −5), so no protonation took place, producing no mixture modifications. Indeed, all the samples remained solutions, with no appreciable viscosity.

The results are very different at pH 5 when Boc-L-Dopa(Bn)_2_-OH was added at a 1% *w*/*v* concentration (condition **B**). Under these conditions, gelation occurred for samples **1B**–**8B**, and this outcome confirms the strong gelation ability of the gelator, when its carboxylate group is protonated, even in complex mixtures that in principle could interfere with the gelification process.

**Figure 4:**
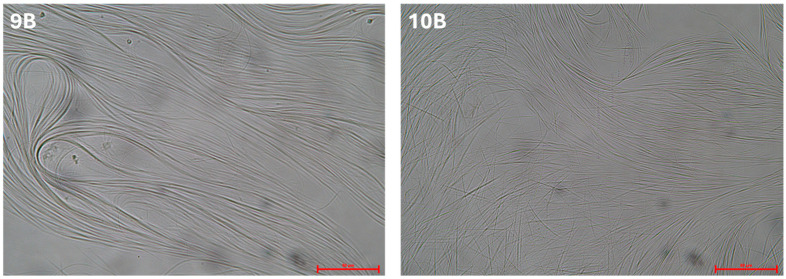
Optical microscope images (40× magnification) of samples 9B (**left**) and 10B (**right**). The scalebar is 50 µm.

The samples containing surfactants 9 and 10 show a clear transformation upon the addition of a gelator (Figure 3), even though they remain in the liquid state. Nevertheless, they change from being completely transparent (**9A** and **10A**) to white (**9B** and **10B**), indicating the formation of supramolecular structures large enough to scatter visible light. This suggests a reorganization at the microscopic level, leading to a new structural arrangement in the system that creates a 3D network unable to immobilize the solvent, but still causes an increase in the viscosity. The formation of supramolecular structures is confirmed by optical microscope images (Figure 4), showing the presence of long fibers.

Then, CAPB at a 4.5% *w*/*v* concentration was added to the mixture (condition C) and a new set of samples was prepared (**1C**–**10C**). The addition of CAPB has a low impact on the appearance of the samples since they seem to be gels, except for 5C upon visual inspection, as clearly shown in Figure 3.

The rheological analysis provided a detailed characterization of the samples’ mechanical properties, confirming or refuting gel formation and offering insights into material strength and elasticity. The storage modulus (G′) represents the elastic response, while the loss modulus (G″) represents the viscous response, and a gel state is indicated when G′ exceeds G″ [48,49]. Amplitude sweep measurements were performed on all samples characterized by self-supporting abilities (support their own weight when inverted).

**Figure 5:**
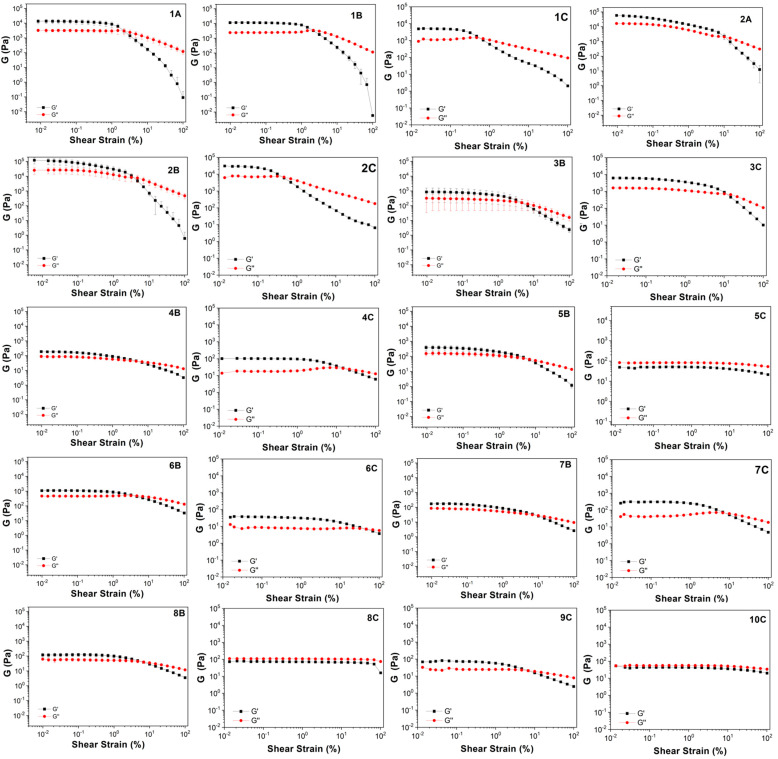
Amplitude sweep test of the 20 gels obtained by gelation procedures of A, B, and C with the ten surfactants studied in this work. Oscillatory amplitude sweep experiments were performed at 25 °C using a Peltier control system and the data points were collected (γ: 0.01–100%) using a constant angular frequency of 1 Hz.

The results obtained with the rheological analysis are shown in Figure 5 and Table 2. Samples **1A** and **2A** are strong gels with a G′ modulus value of about 10^4^ Pa. The addition of Boc-L-Dopa(Bn)_2_-OH (samples **1B** and **2B**) does not significantly modify the results, as **1B** is slightly weaker than **1A**, while **2B** is slightly stronger than **2A**. The results obtained with the addition of CAPB are very similar, with a small decrease in the G′ value for both samples **1C** and **2C**. These results confirm that *N*-substituted amino acid surfactants **1** and **2** may behave as good gelators alone or in mixtures at pH 5.

Regarding surfactants **3**–**10**, the addition of only 1% Boc-L-Dopa(Bn)_2_-OH results in the formation of self-supporting materials in six samples out of eight, and this result is confirmed by amplitude sweep analyses, as G′ is higher than G′′, although often the strength is reduced as G′ never overtakes values of 10^3^ Pascal. In these samples, there is not a synergistic effect between the surfactant and the gelator as previously found because the starting surfactant solutions are completely liquid, so the sample strength is entirely due to the gelator effect, and this accounts for the reduced mechanical properties.

Additionally, when the same formulations are prepared by adding CAPB to the mixture to better mimic a cosmetic formulation, all samples appear to be self-supporting gels, although generally the G′ value decreases, overtaking 10^2^ Pascal only for samples **3C** and **7C**, while for several samples (**5C**, **6C**, **8C**, **9C**, and **10C**), the elastic modulus is less than 100 Pa. Moreover, in the case of **5C**, **8C**, and **10C** the G″ value is slightly higher than G′, so these samples should be more correctly defined as viscous liquids and not gels, although their appearance is very similar and they can still be useful for a variety of cosmetic products.

To explain this outcome, the complex viscosity (η*) of the three samples was also reported. In all cases, the values of the C samples are very close to the values of the B samples, thus showing that all these samples are borderline between gels (G′ > G″) and viscous liquid (G″ > G′). Nonetheless, it can be stated that in any case, the addition of the gelator highly affects the rheology of the surfactant solutions, converting almost all of the samples from liquids to gels or viscous liquids, increasing the viscosity.

Frequency sweep analyses (Figure S1) further elucidate the viscoelastic nature of the samples. In most cases reported in Figure S1, the G′ value exceeds G″, even if the two are close and show a frequency dependence, thus reflecting a gel-like behavior, as previously demonstrated by Winters and coworkers [50,51,52,53]. Sample 8C is the only exception, so it should be considered a viscous liquid instead of a proper gel.

The samples were also observed using an optical microscope (Figure S2) to better characterize the structures formed. Surfactants 1 and 2, which can form gels on their own, show the presence of fibrous aggregates. In both cases, the formed networks are greatly affected by the addition of the gelator and the amphoteric surfactant in terms of fiber density, shape, and size.

For most of the samples, there is a general trend indicating that the use of anionic surfactants and gelators (sample B) leads to the formation of larger fibrous structures, characteristic of the Boc-L-Dopa(Bn)_2_-OH gelator, compared to the network observed in the presence of CAPB (samples C). This structural difference correlates with the rheological data that show stronger mechanical properties in the presence of larger fibers as observed in samples **4B**, **5B**, **6B**, and **8B**, compared to the corresponding C samples. In contrast, sample **7C** shows a larger fiber size compared to **7B**, due to the presence of CAPB, causing increased mechanical properties.

## 3. Conclusions

This manuscript details the preparation and analysis of thirty samples containing commercial surfactants commonly used in cosmetic formulations at pH 5, which is the physiological pH of the human skin and scalp. This study investigates the impact of adding a small percentage of an efficient low-molecular-weight gelator (LMWG) on the appearance and consistency of these surfactant mixtures, focusing on its potential as a rheological modifier and viscosity enhancer. Specifically, 1% *w*/*v* of the LMWG Boc-L-Dopa(Bn)_2_-OH was added to two sets of aqueous solutions: one containing ten different anionic surfactants (10% *w*/*v*) and the other containing the same anionic surfactants plus cocamidopropyl betaine (4.5% *w*/*v*), a zwitterionic surfactant known for its mildness, to better mimic a cosmetic formulation. In the majority of cases, the addition of just 1% of the gelator induced the formation of fibers, demonstrating its ability to self-assemble at pH 5, even within these complex mixtures at a high concentration of surfactants. The rheological analysis (amplitude sweep and frequency sweep) confirmed the gel-like behavior of most of the samples, with the storage modulus (G′) exceeding the loss modulus (G″). Other samples that seemed to be gel upon visual inspection and from the inversion of the vial were instead revealed to be viscous liquids, which can still be very useful in a variety of applications (detergents and cosmetic formulations).

This preliminary work suggests a promising new approach to developing more sustainable cosmetic formulations by replacing synthetic polymers with versatile and biodegradable LMWGs. This substitution addresses growing concerns about the environmental impact of the microplastics and non-biodegradable ingredients commonly used in cosmetics. By leveraging the unique properties of LMWGs, such as their ability to form diverse structures and their inherent biodegradability, we aim to create cosmetic products with improved sustainability profiles without compromising performance or sensory attributes. Further research will focus on optimizing the formulation, for example by changing the gelator percentage to modify its rheological behavior and characterizing the long-term stability and efficacy of these LMWG-based cosmetic products, paving the way for wider adoption of bio-based materials in the personal care industry. This approach can also be implemented in standard manufacturing facilities because it requires pH commonly used in implants and mixing systems. Additionally, the large-scale synthesis of LMWGs can be outsourced to companies specialized in custom peptide synthesis, making the process feasible for industrial application.

## 4. Materials and Methods

The anionic surfactant Eversoft™ ACS-30S was purchased from Sino Lion (Florham Park (NJ) USA); Galsoft SCG was purchased from Galaxy Surfactants Ltd (Navi Mumbai, India).; Pureact WS Conc, Iselux^®,^ and NANSA^®^ LSS 38/AV were purchased from Innospec Performance Chemicals (Salisbury (NC) USA); STEPAN-MILD^®^ PCL-BA was purchased from STEPAN EUROPE S.A.S (Voreppe, France); PROTELAN AGL 95, PROTELAN LS 9011/SL, SETACIN 103 SPEZIAL NP, and ZETESOL MG-FS were purchased from Zschimmer & Schwarz Italiana S.p.A. (Tricerro (VC) Italy). All the solvents were purchased from Sigma-Aldrich (St. Louis, MO, USA). Boc-L-Dopa(Bn)_2_-OH was synthesized following a multistep procedure in the solution as reported in the literature [38,54] (Scheme S1).

The pH meter XS pH 8 PRO Basic (XS Instruments, Carpi (MO), Italy) equipped with XS Sensor Standard T BNC was used to measure the samples’ pH levels.

The rheological analyses were performed using an Anton Paar (Graz, Austria) MCR 92 rheometer. A cone (d = 50 mm)/plane measuring system was used, setting a gap of 0.098 mm. Oscillatory amplitude sweep experiments were performed at 25 °C using a Peltier control system, and the data points were collected (γ: 0.01–100%) using a constant angular frequency of 1 Hz. Frequency sweep experiments were performed at γ = 0.01%, ω = 0.1–10 rad/s, and T = 25 °C.

- Optical microscopy. The images were recorded using a Nikon (Tokyo, Japan) Eclipse 90i optical microscope and a 40× magnifier. The gels used for optical microscopy were prepared as described, and then a small piece was transferred onto a glass microscope slide and analyzed.

- Gel preparation. Each surfactant in the study has a specific concentration range. For this reason, different amounts were withdrawn with a pipette or weighed using an analytical balance to achieve 10% active matter in the solution (Table S1). Cosmetic raw materials generally allow for a relatively wide range within their specification limits. The average value of the active matter, reported below (% *w*/*v*), was selected to determine the amount of raw material to use in each test. Each surfactant was assigned a number from 1 to 10:

- Sample A contained 10% surfactant.
- Sample B contained 10% surfactant + 1% Boc-L-DOPA(Bn)_2_-OH (equivalent to 0.105 mmol for 5 mL of the sample).
- Sample C contained 10% surfactant + 1% Boc-L-DOPA(Bn)_2_-OH and 4.5% Cocamidopropyl betaine.

- Details for the preparation of **sample A**, with surfactants **1**–**10**. All samples were prepared inside 10 mL glass squat vials with a total volume of 5 mL. The surfactant (10%) and water were mixed with a magnetic stirrer for 15 min. Lactic acid (45%) was added to reach a final pH of 5 while mixing with a magnetic stirrer since simple swirling leads to non-homogeneous samples. Only in the case of Zetesol MG-FS (sample **8A**) was the initial pH too low (4.5) so no acid was added, and NaOH was used instead to bring the pH to the final value of 5.

- Details for the preparation of sample B, with surfactants 1–10 and Boc-L-Dopa(Bn)2-OH. All samples were prepared inside 10 mL glass squat vials with a total volume of 5 mL. The gelator (1.0% *w*/*v*) was suspended in osmotic H2O with the surfactant (10% *w*/*v*) and a volume of NaOH (7.5% *w*/*v*) to reach pH 10. The mixture was stirred and sonicated until the complete solubilization of the gelator. Lactic acid (45%) was added to reach a final pH of 5 while mixing with a magnetic stirrer for 1 min since simple swirling leads to non-homogeneous samples.

- Details for the preparation of sample C, with surfactants 1–10, Boc-L-Dopa(Bn)2-OH, and CAPB. All samples were prepared inside 10 mL glass squat vials with a total volume of 5 mL. The gelator (1.0% *w*/*v*) was suspended in osmotic H2O with the surfactant (10% *w*/*v*) and a volume of NaOH (7.5% *w*/*v*) to reach a starting pH of 10. The mixture was stirred and sonicated until the complete solubilization of the gelator, and after that, the amphoteric surfactant CAPB (Cocamidopropyl betaine, 4.5% *w*/*v*) was added. Lactic acid (45%) was added, reaching a final pH of 4.2. Mixing was achieved by swirling for liquid samples and using a spatula for the more viscous ones.

**Figure 6:**
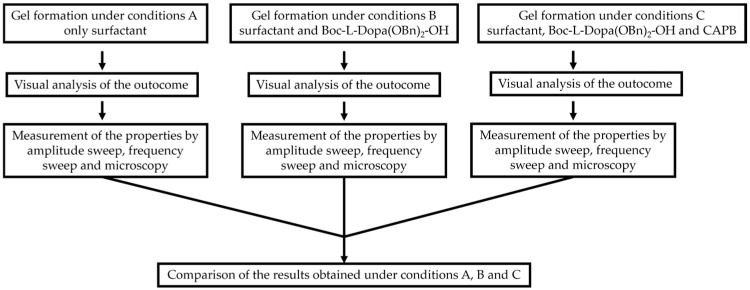
Flow chart of the experimental procedures.

The flow chart representation of the experimental procedures is reported in Figure 6.

## Figures and Tables

**Table 1:** List of the surfactants described in this work. The reported chemical structures of compounds **1**–**10** show the structure of the main component of each sample.

Trade Name	Class	Number	Chemical Structure
EVERSOFT^TM^ ACS	*Alaninate*	**1**	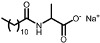
GALSOFT SCG	*Glycinate*	**2**	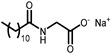
PROTELAN AGL 95	*Akylglutamate*	**3**	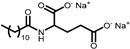
PROTELAN LS 9011/SL	*Sarcosinate*	**4**	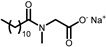
PUREACT WS CONC	*Taurate*	**5**	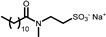
STEPAN MILD^®^ *PCL*	*Sulphonate*	**6**	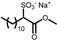
ISELUX^®^	*Alkylmethyl isothionate*	**7**	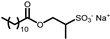
ZETESOL MG FS	*Alkylsulphate*	**8**	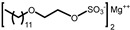
SETACIN 103 SPEZIAL	*Solphosuccinate*	**9**	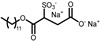
NANSA^®^ LSS 38 AV	*Olefinesulphonate*	**10**	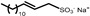

**Table 2:** Details of the results obtained for the rheological analyses of samples described in this work. All the recorded data were collected from the oscillatory amplitude sweep analyses, at γ = 0.03%, setting a constant temperature of 25 °C and a constant angular frequency of 1 Hz.

Sample	G′ (Pa)	G″ (Pa)	tanδ	G*	η* (Pa)
**1A**	13,426.3	3207.2	0.248	13,811.7	2198.2
**1B**	11,300.7	2420.4	0.215	11,557.0	1839.4
**1C**	5037.4	1105.7	0.219	5157.3	820.8
**2A**	49,688.7	15,778.3	0.317	52,134.0	8297.4
**2B**	103,926.3	26,462.0	0.267	107,256.3	17,070.6
**2C**	29,747.0	7826.8	0.263	30,760.0	4895.6
**3B**	838.6	296.6	0.365	889.9	141.6
**3C**	6179.6	1597.4	0.258	6382.7	1015.8
**4B**	180.0	86.0	0.478	199.5	31.8
**4C**	105.4	18.5	0.175	107.0	17.0
**5B**	376.3	153.8	0.421	406.7	64.7
**5C**	44.0	81.7	1.856	92.8	14.8
**6B**	1094.6	457.2	0.424	1187.3	189.0
**6C**	38.1	7.4	0.195	38.8	6.2
**7B**	175.8	79.9	0.458	193.2	30.7
**7C**	312.9	42.7	0.136	315.8	50.3
**8B**	118.8	57.2	0.494	132.1	21.0
**8C**	77.6	110.3	1.421	134.9	21.5
**9C**	74.5	23.2	0.312	78.1	12.4
**10C**	39.3	57.7	1.468	69.9	11.1

## Data Availability

The original contributions presented in this study are included in the article/Supplementary Material. Further inquiries can be directed to the corresponding authors.

## Supplementary Materials

The following supporting information can be downloaded at: https://www.mdpi.com/article/10.3390/gels11050320/s1, Scheme S1. Scheme of the synthesis of Boc-L-DOPA(Bn)_2_-OH, Table S1. Commercial surfactants used, Figure S1. Photographs of gelation of samples 1A-10A, Figure S2. Photographs of gelation of samples 1B-10B.

## Abbreviations

The following abbreviations are used in this manuscript:

Boc-L-Dopa(Bn)_2_-OH	(*S*)-*N*-(*tert*-butyloxycarbonyl)-3,4-bis(benzyloxy)-phenylalanine
LMWG	Low molecular weight gelator
CAPB	Cocamidopropyl betaine
AAS	*N*-substituted Amino Acid Surfactant
