# Supplementary material for: Application of a Dopa Derivative for the Formation of Gels in the Presence of Commercial Surfactants
Source: Gels. 2025 Apr 25;11(5):320. doi: 10.3390/gels11050320 (PMC12111070; doi:10.3390/gels11050320)
Supplement: Supplementary file 1 [file gels-11-00320-s001.zip › gels-3524821-supplementary.pdf]

SUPPORTING INFORMATION

Application of a Dopa Derivative for the Formation of Gels in the Presence of Commercial Surfactants

Sofia Chinelli, Fabia Cenciarelli, Demetra Giuri * and Claudia Tomasini *

Dipartimento di Chimica Giacomo Ciamician, Università di Bologna, Via Piero Gobetti, 85, 40129 Bologna, Italy; sofia.chinelli2@unibo.it (S.C.); fabia.cenciarelli2@unibo.it (F.C.)
* Correspondence: demetra.giuri2@unibo.it (D.G.); claudia.tomasini@unibo.it (C.T.)

**Scheme S1.** Scheme of the Synthesis of Boc-L-DOPA(Bn)$_2$-OH	Page S2
**Table S1.** Commercial surfactants used	Page S3
**Figure S1.** Frequency sweep analyses of the samples obtained (γ=0.01%, T=25°C).	Page S4
**Figure S2.** Optical microscope images of the samples obtained. The scalebar represents 50 μm.	Page S5-S6

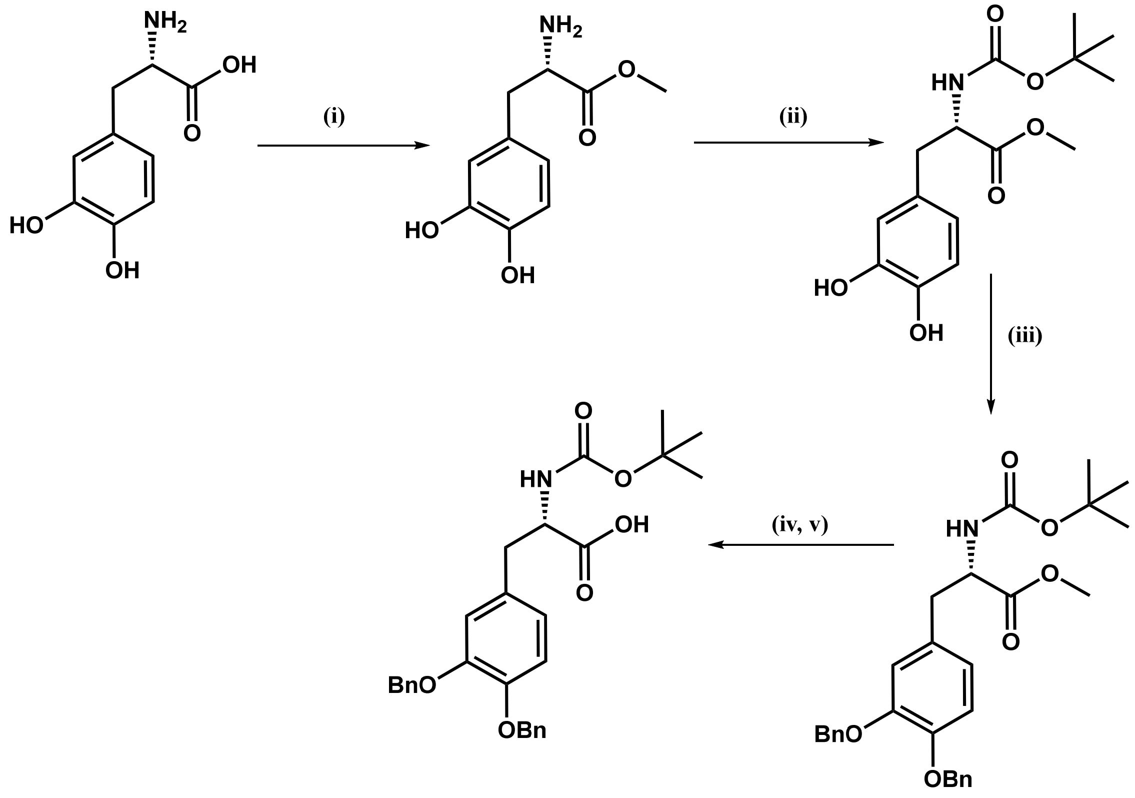

Scheme S1. Reagents and conditions: (i) L-DOPA-OH (1 equiv.), $SOCl_2$, MeOH; (ii) Boc_2O (2 equiv.), $NaHCO_3$ (2 equiv.), THF/H_2O, r.t., 18 h; (iii) BnBr (2.2 equiv.), K_2CO_3 (2.2 equiv.), TBAB (0.2 equiv.), NaI (0.2 equiv.), acetone, reflux, 4 h; (iv) 1 M NaOH, MeOH/THF, r.t., 18 h.

Table S1. Commercial surfactants used.

n.	COMMERCIAL NAME *Surfactants*	Average % of active matter in the raw material	pH	Quantity for 10% active matter in x 5g
**1**	**EVERSOFT™ ACS** *Alalinate*	30.00	9.0	1.67 ml
**2**	**GALSOFT SCG** *Glycinate*	22.00	10.5	2.27 ml
**3**	**PROTELAN AGL 95** *Glutamate*	37.00	9.5	1.35 ml
**4**	**PROTELA LS 9011/SL** *Sarcosinate*	30.00	10.0	1.67 ml
**5**	**PUREACT WS CONC** *Taurate*	30.75	7.9	1.626 g
**6**	**STEPAN MILD®** *Sulphonate*	23.00	6.0	2.17 ml
**7**	**ISELUX®** *Isothionate*	80.00	6.2	0.625 g
**8**	**ZETESOL MG-FS** *Alkylsulphate*	25.00	4.3	2.00 ml
**9**	**SETACIN 103 SPEZIAL** *Solfosuccinate*	39.50	6.5	1.27 ml
**10**	**NANSA® LSS 38 AV** *Olephine sulphonate*	29.00	7.0	1.72 ml

The surfactants used had different initial pH values, which influenced the amount of NaOH needed to adjust the pH and ensure proper solubilization of the gelling agent, as well as the quantities of acid triggers necessary to achieve the target pH.

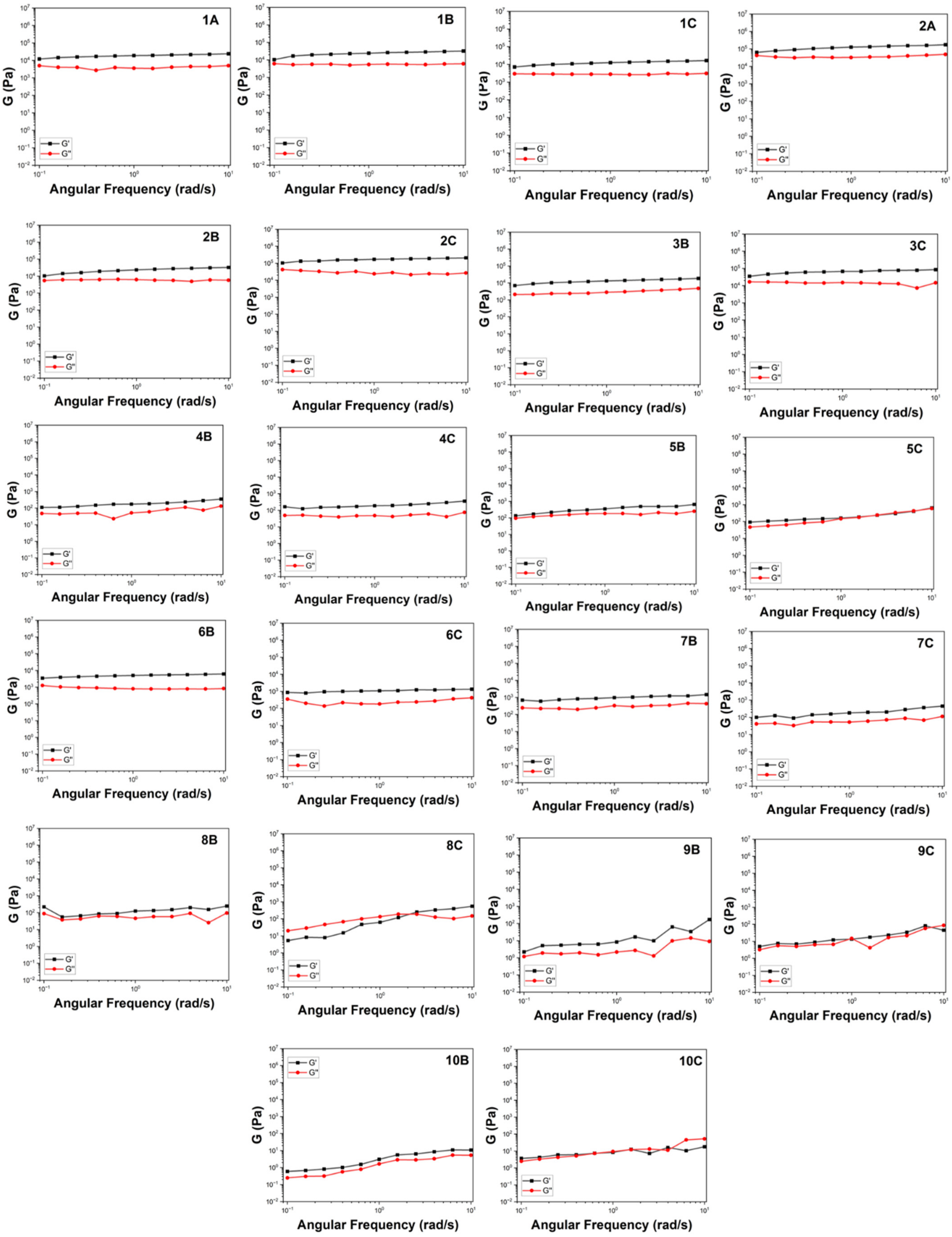

Figure S1. Frequency sweep analyses of the samples obtained (γ=0.01%, T=25°C). The frequency range was limited to 0.1–10 rad/s to avoid artifacts caused by instrument inertia when measuring low-modulus samples at higher frequencies.

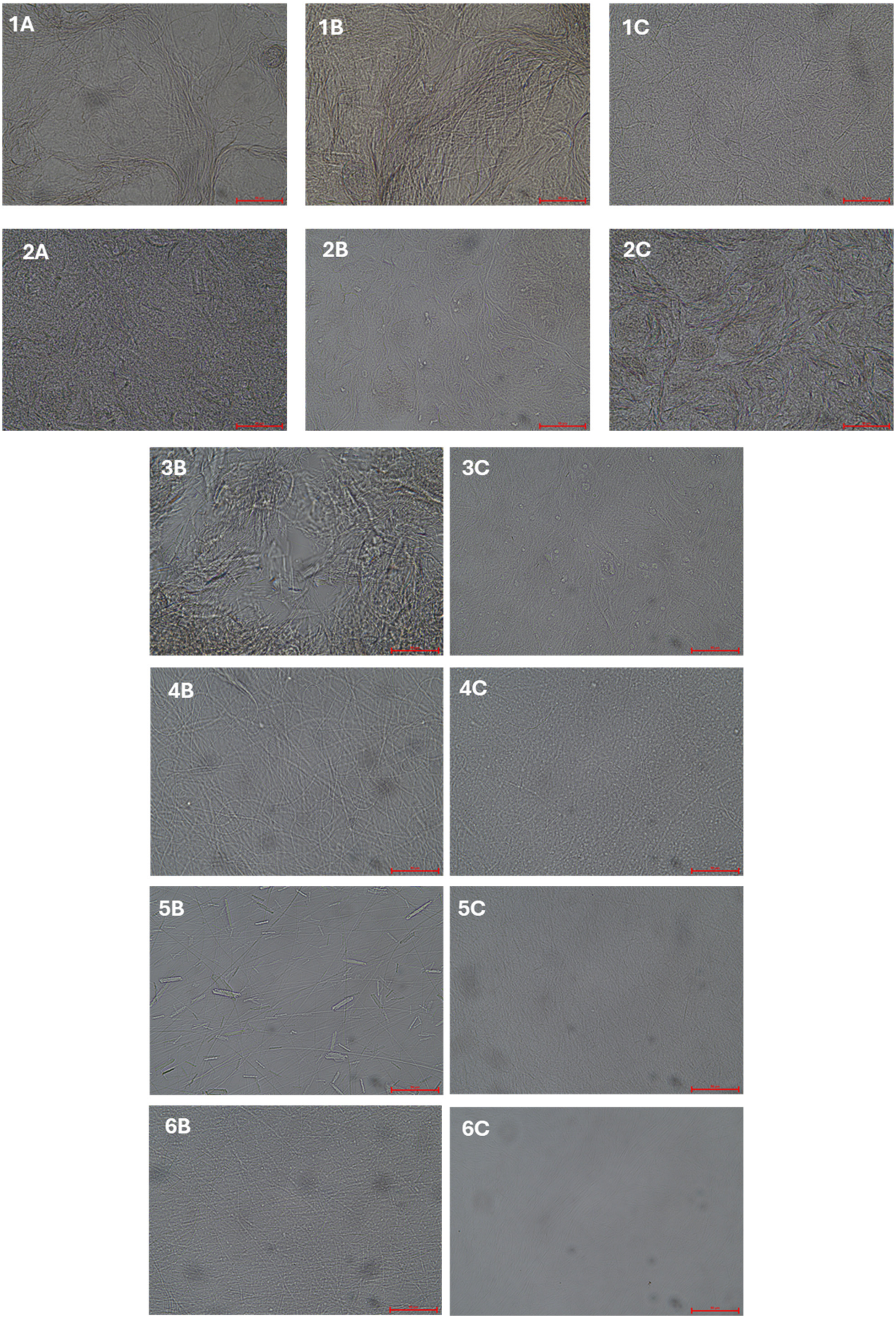
1A
1B
1C
2A
2B
2C
3B
3C
4B
4C
5B
5C
6B
6C

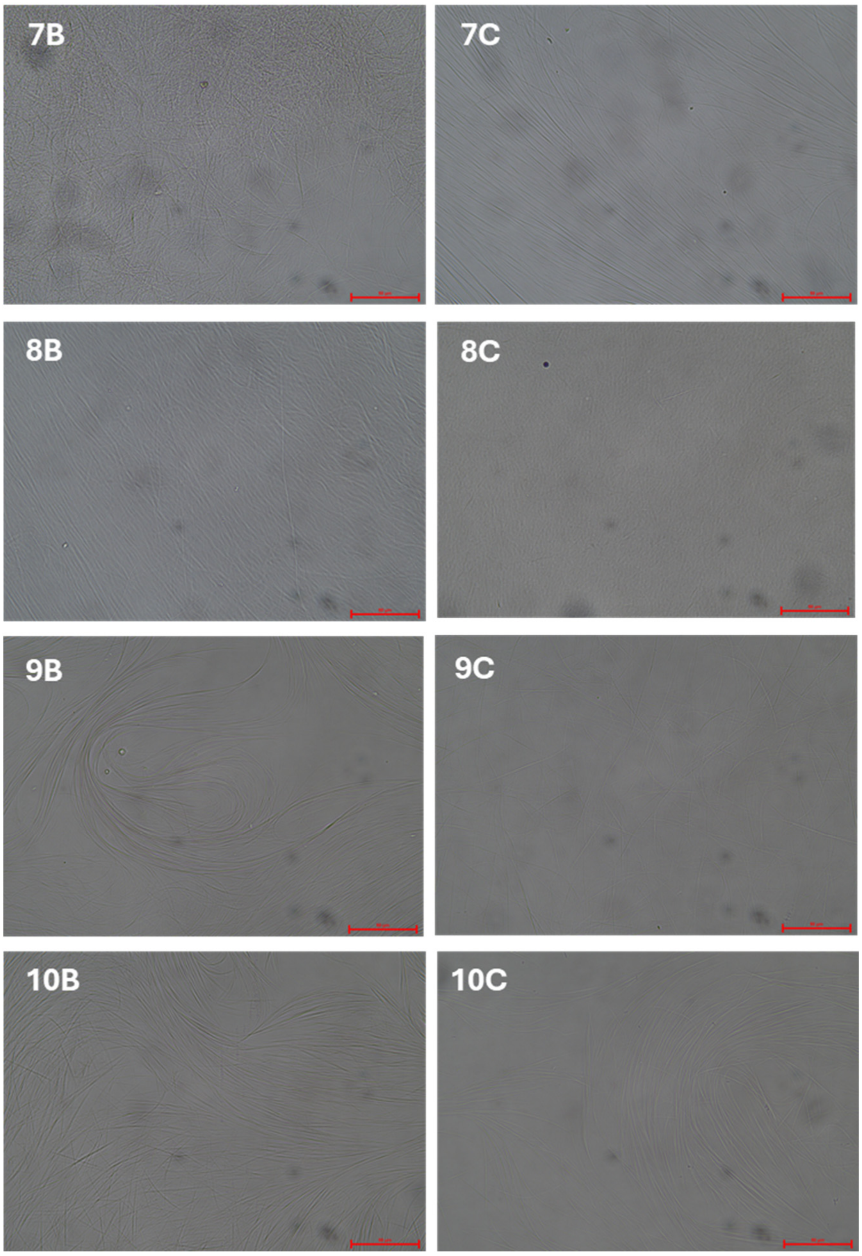

Figure S2. Optical microscope images of the samples obtained. The scalebar represents 50 μm.
